# XAI GNSS—A Comprehensive Study on Signal Quality Assessment of GNSS Disruptions Using Explainable AI Technique

**DOI:** 10.3390/s24248039

**Published:** 2024-12-17

**Authors:** Arul Elango, Rene Jr. Landry

**Affiliations:** 1 Vignan’s Foundation for Science, Technology and Research, Guntur 522213, Andhra Pradesh, India; 2 LASSENA—Laboratory of Space Technologies, Embedded Systems, Navigation and Avionics, École de Technologie Supérieure (ETS), Montreal, QC H3C-1K3, Canada; renejr.landry@etsmtl.ca

**Keywords:** GNSS, jamming, explainable AI, interpretability, interference

## Abstract

The hindering of Global Navigation Satellite Systems (GNSS) signal reception by jamming and spoofing attacks degrades the signal quality. Careful attention needs to be paid when post-processing the signal under these circumstances before feeding the signal into the GNSS receiver’s post-processing stage. The identification of the time domain statistical attributes and the spectral domain characteristics play a vital role in analyzing the behaviour of the signal characteristics under various kinds of jamming attacks, spoofing attacks, and multipath scenarios. In this paper, the signal records of five disruptions (pure, continuous wave interference (CWI), multi-tone continuous wave interference (MCWI), multipath (MP), spoofing, pulse, and chirp) are examined, and the most influential features in both the time and frequency domains are identified with the help of explainable AI (XAI) models. Different Machine learning (ML) techniques were employed to assess the importance of the features to the model’s prediction. From the statistical analysis, it has been observed that the usage of the SHapley Additive exPlanations (SHAP) and local interpretable model-agnostic explanations (LIME) models in GNSS signals to test the types of disruption in unknown GNSS signals, using only the best-correlated and most important features in the training phase, provided a better classification accuracy in signal prediction compared to traditional feature selection methods. This XAI model reveals the black-box ML model’s output prediction and provides a clear explanation of the specific signal occurrences based on the individual feature contributions. By using this black-box revealer, we can easily analyze the behaviour of the GNSS ground-station signals and employ fault detection and resilience diagnosis in GNSS post-processing.

## 1. Introduction

The positioning-based applications available today that rely on satellite navigation systems, including the widely used Global Positioning System (GPS), Russian GLONASS, European GALILEO, Chinese BEIDOU, Japanese QZSS and Indian IRNSS, have become integral parts of various aspects of modern life, including transportation, agriculture, telecommunications, and emergency services. These systems depend on a collection of satellites orbiting the globe to provide accurate positioning, navigation, and timing information to users worldwide. However, they are susceptible to interference, which can disrupt the signals and compromise their reliability. Satellite navigation interference refers to deliberate or unintentional disruptions of signals between satellites and ground-based receivers. Interference can take various forms, including jamming, which is the deliberate transmission of strong radio signals on the same frequencies used by satellite navigation systems to overpower and disrupt genuine satellite signals. The jamming devices are sometimes used for illegal purposes, for example, the usage of cheap commercial hardware jammers, such as cigarette lighters, available on the market. Other illegitimate GNSS creations, like signals erupting from high-power transmitters, also degrade the quality of the signal by completely blocking the signal reception and not allowing the GNSS satellites to compute the user position. Another kind of attack is spoofing, which is a more sophisticated form of interference where attackers generate fake satellite signals that mimic authentic ones. Spoofing can deceive receivers into calculating inaccurate positions. Signal blockage may be natural or deliberate; for example, obstructions caused by human intervention and obstacles created by the construction of tall buildings or terrain can block or reflect satellite signals, causing multipath errors that lead to inaccuracies in positioning. Multipath interference signals bouncing off nearby surfaces before reaching the receiver can create timing errors and inaccuracies.

In the event of GNSS catastrophic failures, continuous monitoring and taking precautionary measures to detect unauthorized jamming and spoofing are required to prevent failures in GNSS data transmission at ground stations [1,2,3]. Early detection and rapid responses to counteract the impact of disruptions are required for seamless data reception and transmission [4,5]. Recently, some solutions have involved implementing security protocols and GNSS signal encryption techniques in navigation data to resist these kinds of interruptions, which could make it more difficult to disrupt or manipulate the signals. In the GNSS-denied situation, alternative navigation techniques, like the use of inertial measurement units (IMUs), a mixed-mode operation that involves using accelerometers and gyroscope fusion, may help to measure the acceleration and angular velocity, which enhances the resilience and redundancy. Subsequently, computed features from the data serve as input to the ML and deep learning (DL) techniques for classifying and detecting the types of interruption. However, there is no proper explanation in the model output that describes how the model is responding to the changes in the signal and what factors influence the identification of key discriminative factors in distinguishing different classes of disruptions.

Taking into consideration all the above shortcomings in signal detection using traditional ML techniques, this study proposes a novel framework for analyzing the GNSS signal characteristics. The proposed approach not only enables the accurate classification of GNSS signal disruptions but also provides valuable insights into the underlying factors driving these disruptions. The main contributions of the work are summarized as follows:

1. In this paper, we recorded a synthetic GNSS dataset under various disruptions at different Jamming-to-Signal Ratio (JSR) levels using the Skydel simulator. The dataset was used to evaluate the performances of different ML models on signal prediction/classification tasks.
2. We extracted and compared a set of attributes (time domain and frequency domain) using the statistical parameters to identify the strongly and weakly correlated features, as visualized in the correlation plot.
3. We utilized the most influential features from the global (SHAP) and local explanation (LIME) XAI models in the form of feature rankings, a summary plot, and a forced plot with detailed explanations, using only the key features for predicting and classifying the signal disruption resulted in improved metrics.

## 2. Literature Review

There are some existing approaches based on the artificial intelligence applied in GNSSs that are discussed in articles that describe the utilization of different ML/DL models at different stages, such as the RF front-end stage, pre-correlation stage, and post-correlation stage [6,7,8,9,10,11,12,13,14]. The different ML algorithms used in the literature, such as the distance-based representational model, autoregressive integrated moving average (ARIMA), and statistical measures (mean, standard deviation, variance, median, quantile, kurtosis, skewness, etc.), have been shown that they have several shortcomings and that they cannot be used to model multivariate time series data. Some of the traditional statistical approaches used in [15] provided information about the trends and outliers in the signal, with the help of the correlation in the data on 30% of the quartile error values. Many categories of anomalies and several ML algorithms listed in the literature (supervised, semi-supervised, and unsupervised) were used to detect the GNSS abnormalities in the signal. A very detailed description of the type of data, the categories of anomalies, and the evaluation criteria are provided in [16] in the context of Internet of Things (IOT) data, which paved the way for analyzing the suitability of the selected deep learning model. Similarly, a survey of ML models and a variety of ML-based abnormality detection schemes illustrated in [17] created an interest among readers in choosing the best model for anomaly detection.

Some of the studies listed here used real- and complex-valued long short-term memory (LSTM) algorithms that predicted the next time steps based on loss values [18,19]. A hyper approach developed based on LSTM and a one-class support vector machine-based anomaly detection method has been implemented by Elsayed et al. [20] for detecting attacks in the network of an unbalanced dataset. The method employed has the advantage of being able to operate on high-dimensional datasets by reducing the processing time. Using this hybrid model, a high detection rate was obtained for securing the Software Defined Network (SDN) from malicious traffic. An LSTM network layer has been used to denoise and forecast the interruption in the GNSS permanent station for early warning monitoring on different time series data [21,22]. However, the data used in this work rely on post-processing observations. A recurrent neural network (RNN)-based autoencoder network constructed by Wu et al. [23] is a good choice for learning the bonds among time-correlated data. This autoencoder network could detect interference with F1-scores of 0.93, 0.90, and 0.83, respectively, under three communication scenarios: a 16-quadrature amplitude modulated (QAM) signal with superimposed quadrature phase shift keying (QPSK), a frequency modulated (FM) signal, and a clean FM signal contaminated by a direct sequence spread spectrum (DSSS). This model only considers anomaly detection in generic modulation schemes.

An overview of the use of LSTM models in GNSS jamming and spoofing and their limitations is presented next. Spoofing detection is carried out with the help of a general adversarial network (GAN) by taking the cross-ambiguity function (CAF) as an acquisition output for both authentic and spoofed signals [24]. The acquisition of satellite signals needs to be performed as a function of receiver signal processing. A jamming detection method using an LSTM-based model was introduced by [25] for satellite signal reception in the presence of alpha-state noise. The pure satellite signal of interest that is not a jamming instance is used for LSTM training. Here, the myriad filter was employed to suppress the alpha-state noise. A detection rate of 98% was achieved for a JSR of 7 dB. The random motion between the transmitted and received GPS signals differs from the live sky-generated Doppler patterns that are used to find the presence of abnormal behaviour in the Doppler frequency or malicious attackers to analyze the spoofing threats in GNSSs. In another study [26], a training dataset obtained from the daily live record data uploaded by NASA was used to train the LSTM models for spoofing detection. The architecture implemented consisted of two LSTM layers with 32 neurons, with one dropout layer and one dense layer, for predicting the spoofing attacks. The Doppler shift detected from the post-processing of the GPS signal is used for training. The hybrid learning framework of GNSS observations using anomaly detection via the hierarchical density-based spatial clustering of applications with noise (HDBSCAN) technique has been used by Xia et al. [27]. The ComNav K508 GNSS OEM receiver was used to log the Receiver INdependent EXchange (RINEX) data that are used for training purposes, and the NovAtel ProPak6 receiver was used to collect the test set for real-time anomaly detection. Anomaly detection in the transponder frequency spectra of satellite communications systems with high-dimensional time series data using LSTM networks is implemented by Gunn et al. [28]; this provides a lower reconstruction error, but the time-frequency approach for collecting the images for each snapshot increases the data handling and the data storage complexity. A machine learning-based identification of the clean, non-line of sight (NLOS), and multipath signals in a GNSS is presented in [29]. The variations in the received power level, the pseudorange residue, and changes in the variance between the pseudorange and delta pseudorange obtained from the National Electrical Manufacturers Association (NEMA) and RINEX are used to train the machine learning algorithm.

To handle the time synchronization attacks, a three-layer multi-layer perceptron neural network was implemented in [30] to nullify jamming in stationary GPS receivers. The authors prepared the clock offset dataset of 200 samples used as an input to the model for training, with different threshold conditions that lead to spoofing detection and positioning error reduction. Spoofing detection in GNSS signals with the aid of the LSTM prediction model has been implemented by Dasgupta et al. [31] using a publicly available real-world driving dataset. The data captured from the controlled area network (CAN), GNSS, and IMU sensors with different parameters such as acceleration, steering wheel angle, speed, and distance are used to detect spoofing attacks in autonomous vehicles.

To the best of our knowledge, most of the methods listed above used either traditional statistical methods [32,33] or spectrogram images, post-processed data, correlation outputs, and data collected at the navigation level to train an ML/DL model to identify possible trends in the signal quality and abnormalities in GNSS data. However, there is still a need to determine the reason behind these changes in the signal behaviour, and the cause of abnormalities detected at the RF signal level has to be identified before the signal is fed into the receiver signal processing chain. Nevertheless, the aforementioned literature studies used several ML/DL models and collected the most influential features by suitably classifying or predicting the anomalous GNSS signals with proper justification. However, their AI models failed to provide the reasons behind the classification/prediction and how the classification decisions were made. Also, it is hard to know on what basis the AI model is functioning and what aspects the model uses to determine the result. Because of the lack of transparency and trustworthiness of these AI models, they are called black-box models, and therefore, it is necessary to know the causes and effects of signal disruption or what would happen if the signal attributes changed.

To solve this problem, XAI methods are used. These methods are very popular in the AI community and very well-known for making decisions and understanding or detecting the root cause of a correct/wrong prediction or classification in terms of all aspects [34,35,36,37,38]. XAI algorithms such as LIME and SHAP gained more attention because they can provide an explanation of the prediction results of a variety of applications [39,40,41,42,43]. The pre-ad hoc and post-ad hoc AI models mentioned in the literature, such as the saliency map [44], Grad-CAM [45], DeepLIFT [46], and LRP [47], are applied in ML algorithms to provide more informativeness and faithfulness in medical diagnostics and decision-making processes in safety-critical autonomous systems [48]. To avoid potential failures in the GNSS positioning and timing information, the XAI black-box revealer can be applied to retain the resilience of Position, Navigation, and Timing (PNT)-dependent seamless services and operations.

## 3. GNSS Signal **Disruption** Simulation

The GNSS signal provides the position, velocity, and time (PVT) of the end user anywhere on the globe using the satellite measurements of at least four satellites. For this purpose, right-circularly polarized (RHCP) waves are continuously transmitted over three carrier frequencies, L1, L2, and L5 (1575.42 MHz, 1227.6 MHz, and 1176.45 MHz), respectively. The L1 and L2 carrier signals are modulated with binary phase shift keying (BPSK) modulation and pseudorandom noise (PRN) codes known as the C/A code (coarse and acquisition code) and the encrypted P(Y) code (precision code) [49]. The C/A codes are applicable for dual-purpose use in terms of civilian and military applications. In contrast, the P(Y) code is exclusively used by the US military and under the United States Department of Defense’s authorization [49].

### GNSS Signal Quality Monitoring Setup

In this paper, the GNSS signals are recorded using a Skydel simulated satellite signal, represented in the form of in-phase (I) and quadrature-phase (Q) components. These signals are combined using a 2:1 combiner with various types of signal disruption generated by the simulator’s advanced interference tool environment. The RTL-SDR RF front end is utilized to digitize the signals at a specified sampling rate. To record the files, we can specify the sampling frequency, centre frequency, duration of the recording, etc. The default settings are a centre frequency of 1575.42 MHz, a sampling rate of 2.9 MSPS, 1024 samples per frame, and a duration of 300 s. The file format is int16 in the baseband frequency, separated into two .dat files, where each one is int8. The order of the signal recording is as follows: the first file is the in-phase (I) samples, and the second one is the quadrature (Q) samples. The total number of samples is 32,676, acquired in one millisecond, with a bandwidth of 2 MHz [50].

**Figure 1:**
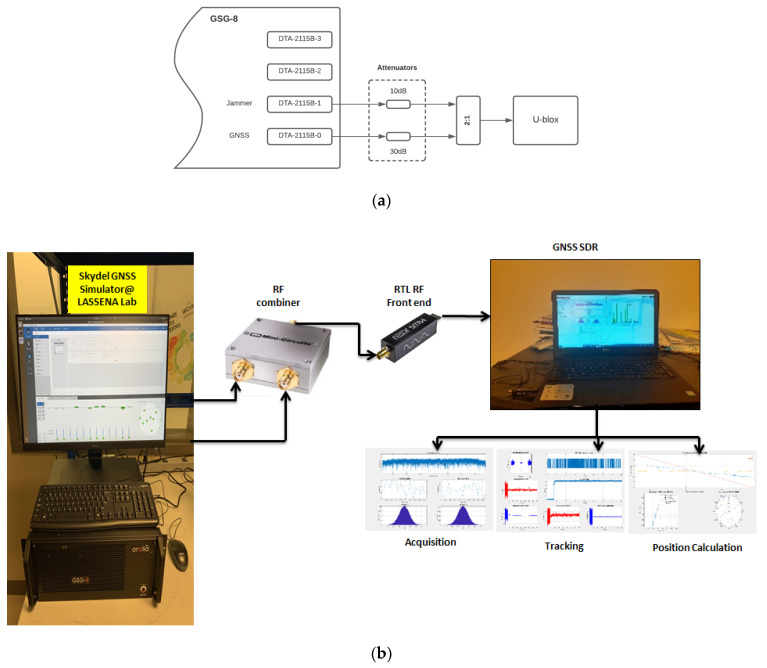
(**a**) Skydel CSG-8 GNSS simulator setup for recording jamming and spoofing signals, (**b**) Various GNSS disruption recording setups and post-processing using GNSS SDR.

The digitized signals are then processed through GNSS receiver modules, including acquisition, tracking, navigation solution, and position calculation. The experimental setup for the GNSS signal under different disruptions is depicted in Figure 1a,b.

**Figure 2:**
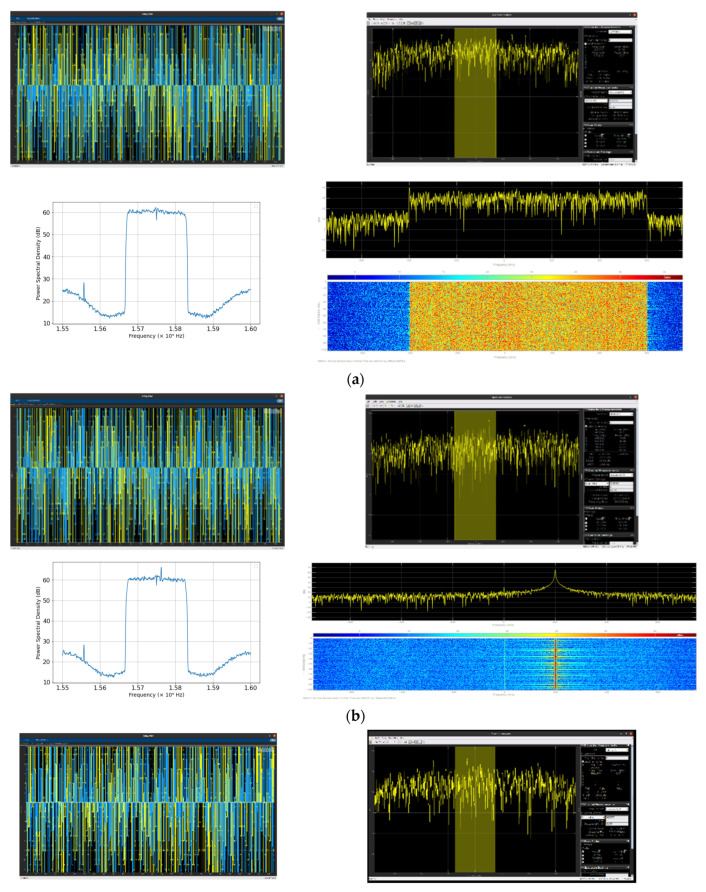

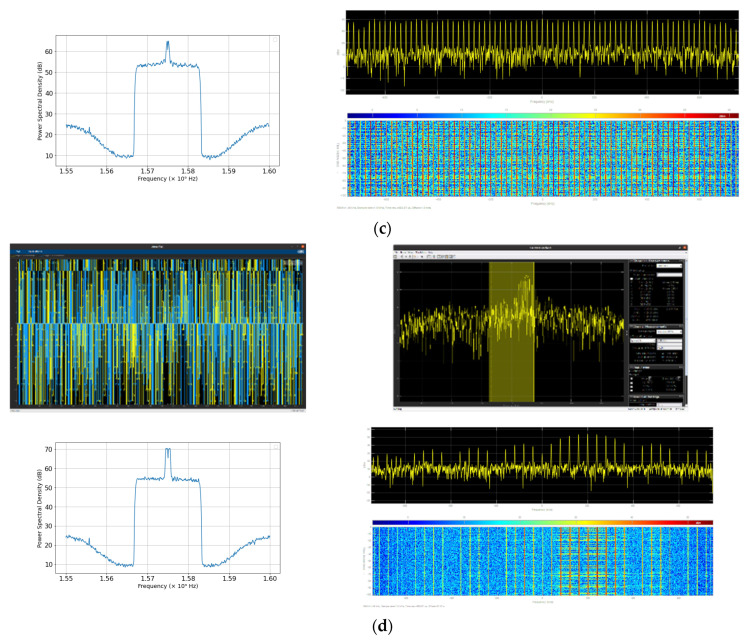
(**a**) Clean GNSS signal in the time domain and its spectrum in the frequency domain recorded using RTL SDR. (**b**) CWI GNSS signals in the time domain and its spectrum in the frequency domain recorded using RTL SDR. (**c**) Pulse interference GNSS signal in the time domain and its spectrum in the frequency domain recorded using RTL SDR. (**d**) Chirp interference GNSS signal in the time domain and its spectrum in the frequency domain recorded using RTL SDR.

The L1 GNSS signal is expressed by

(1) $$S_{L1}=A_{p}\cdot P\left(t\right)\cdot D\left(t\right)\cdot \cos\left(2\pi f_{1}t+\varphi \right)+A_{c}\cdot C\left(t\right)\cdot D\left(t\right)\cdot sin(2\pi f_{1}t+\varphi ),$$

where $S_{L1}$, $A_{p}$, and *P*(*t*) denote the L1 signal frequency, the amplitude, and the phase of the P(Y) code, respectively. *D*(*t*) corresponds to the navigation message, $f_{1}$ is the frequency of the L1 carrier, φ is the initial phase, and $A_{c}\;$and *C*(*t*) are the amplitude and phase of the C/A code, respectively. The nominal GNSS signal in the time domain, its spectrum, and its spectrogram recorded through an RTL SDR are depicted in Figure 2a.

Continuous Wave Interference (CWI)

The CWI-affected GNSS signal is given by

(2) $$CWI=\exp\left(j2\pi f_{cw}t\right),$$

where $f_{cw}$ and *t* represent the centre frequency and period of interference, respectively. The CWI power level is varied from 10 to 60 dB and the centre frequency is set as 1575.42 MHz. Plots of the signal simulation in the time and frequency domains, its spectrum, and the spectrogram recorded for 25 dB can be seen in Figure 2b.

Pulse Interference

The pulse type of interference signal is denoted by

(3) $$PI=\sqrt{P_{j}}\;\;p_{\tau }\left(t\right)⊗\sum_{k=1}^{K}\delta \left(t-\frac{k}{f_{rk}}\right).\exp\left(j2\pi f_{jk}t\right),$$

where $\sqrt{P_{j}}$ and $p_{\tau }\left(t\right)$ are the signal power and the pulse signal of the duty cycle (τ), respectively. $f_{rjk}$ is the pulse repetition frequency, δ is the Dirac pulse, and ⊗ is the convolution operator. Here, the pulse repetition frequency is 10,000 Hz, the duty cycle is 20%, and the signal power is fixed at 25 dB to record the signals in both the time and frequency domains, as shown in Figure 2c.

Chirp Interference (CI)

The mathematical expression for CI is given as follows:

(4) $$CI=\exp(2\pi \frac{k}{2}t^{2}+2\pi f_{0}t).$$

A bandwidth of 10 MHz and a sweeping time of 100 µs are set to record all events under chirp interference, as can be seen from Figure 2d.

## 4. Statistical Quality Assessment of GNSS Signal

In this study, we conducted a comprehensive analysis of GNSS signals by computing both time and frequency domain attributes. The time domain features included the mean, standard deviation, median, mean absolute deviation, root mean square error, 25th and 75th percentiles, inter-percentile range, skewness, kurtosis, entropy, and maximum-to-mean ratio. These time domain features were computed to capture the statistical properties of the signal. These metrics offer clear information about the patterns, central tendency, variability, distribution of power and shape of the spectrum in a specific band of interest, and overall complexity of the signal.

### 4.1. Time Domain Features

The features in the time domain representation are identified based on the statistical parameters computed below:

1. Mean:

The mean value of the samples of the GNSS signal is calculated as

$${Mean}_{j}=\frac{1}{n}\sum_{i=1}^{n}X_{ij}$$

The mean is calculated as the mean value of the ith sample, where$\;X_{ij}$ is the jth feature (or data point) of the ith sample, and n is the number of features (or data points) in each sample.

2. Median:

Similarly, the median value is computed as

$${Median}_{j}=Median\left(X_{i1},X_{i2},\ldots \ldots \ldots X_{in}\right).$$

3. Standard Deviation:

$${Std}_{j}=\sqrt{\frac{1}{n}\sum_{i=1}^{n}{\left(X_{ij}-\mu_{j}\right)}^{2}}$$

4. Mean Absolute Deviation:

$${MAD}_{j}=\frac{\sum_{i=1}^{n}\left|X_{ij}-\mu_{j}\right|}{n}$$

where n is the total number of data points in the dataset.

5. Root Mean Square Error:

$${RMSE}_{j}=\sqrt{\frac{1}{n}\sum_{i=1}^{n}{\left(X_{ij}\right)}^{2}}$$

6. 25th Percentile:

$$P_{25}=percentile\left(X,25\right)$$

7. 75th Percentile:

$$P_{75}=percentile\left(X,75\right)$$

8. Inter-percentile Range:

$${IPR=P}_{75}-P_{25}.$$

9. Skewness:

$${Skewness}_{j}=\frac{\sum_{i=1}^{n}{{(X}_{ij}-\hat{X})}^{3}}{n\sigma^{3}}.$$

10. Kurtosis:

$${Kurtosis}_{j}=\frac{\sum_{i=1}^{n}{{(X}_{ij}-\hat{X})}^{4}}{n\sigma^{4}}.$$

11. Entropy:

$$H_{j}=-\sum_{i}P\left(x_{ij}\right){log}_{2}P\left(x_{ij}\right).$$

12. Maximum-to-Mean Ratio (MTMR):

$${MTMR}_{j}=\frac{\max\left(x_{ij}\right)}{\mu }.$$

Moreover, frequency domain features were calculated to examine spectral characteristics. These include the normalized spectrum bandwidth, normalized spectrum kurtosis (NSK), normalized spectrum flatness (NSF), the ratio of the variance to the squared mean of the normalized spectrum (RVSM), single-frequency energy aggregation, and the ratio of the maximum peak to the second maximum peak of the normalized spectrum (RMPS). These features provide valuable information regarding the distribution of the signal power across different frequency components, a measure of information, the shape of the spectrum concerning the centre frequency, and a measure of the spectral shape and concentration. The spectral domain features are listed in Section 4.2.

### 4.2. Frequency Domain Features

By finding the spectral content of the signal using the fast Fourier transform (FFT), the following attributes were calculated:

1. Normalized Spectrum Bandwidth (NSBW):

$$NSBW=\frac{f_{max}-f_{min}}{f_{c}},$$

where *f_c_* is the centre frequency.

2. Normalized Spectrum Kurtosis (NSK):

$$NSK=\frac{{\sum_{k=f_{1}}^{f_{2}}\left(f_{k}-S_{c}\right)}^{4}P_{k}}{\sum_{k=f_{1}}^{f_{2}}P_{k}s^{4}},$$

where $S_{c}$ is the spectral centroid. It is given by

$$S_{c}=\frac{\sum_{i=1}^{m}f_{k}m_{i}}{\sum_{i=1}^{m}m_{i}}$$

where $f_{1}\;$and $f_{2}$ are the bounds of the frequency band, *f_k_* is the frequency, and $P_{k}$ is the normalized power and m-magnitude of the bin number.

3. Normalized Spectrum Flatness (NSF):

$$NSF=\frac{exp(\frac{1}{N}\sum_{k=1}^{N}ln\left(X(k)\right)}{\frac{1}{N}\sum_{k=1}^{N}\left(X(k)\right)},$$

where X(k) is the magnitude of the k-th frequency bin in the spectrum. N is the total number of frequency bins.

4. Ratio of the Variance to the Squared Mean of the Normalized Spectrum (RVSM):

$$RVSM=\frac{\sigma (X)}{{\left(\sum_{k=1}^{N}X(k)\right)}^{2}},$$

where *X* is the normalized spectrum, σ(X) is the variance of the normalized spectrum, and *N* is the total number of elements in the normalized spectrum.

5. Single-Frequency Energy Aggregation (SFEA):

The summation of the squares of the Fourier transform coefficients results in an energy aggregation given by

$$E_{total}=\sum_{i=1}^{n}{\left|X_{i}\right|}^{2}$$

6. Ratio of the Maximum Peak to the Second Maximum Peak of the Normalised Spectrum (RMPS):

First, find the index in the spectrum $X_{max}$ of the maximum peak in *X*:$\;i_{max}=argmax(X)$ and remove the maximum peak from *X*, denoted as *X*’:$\;X^{\prime }=X/\left\{X\left[i_{max}\right]\right\}$. Then, the second peak is calculated as $max(X^{\prime }$).

**Figure 3:**
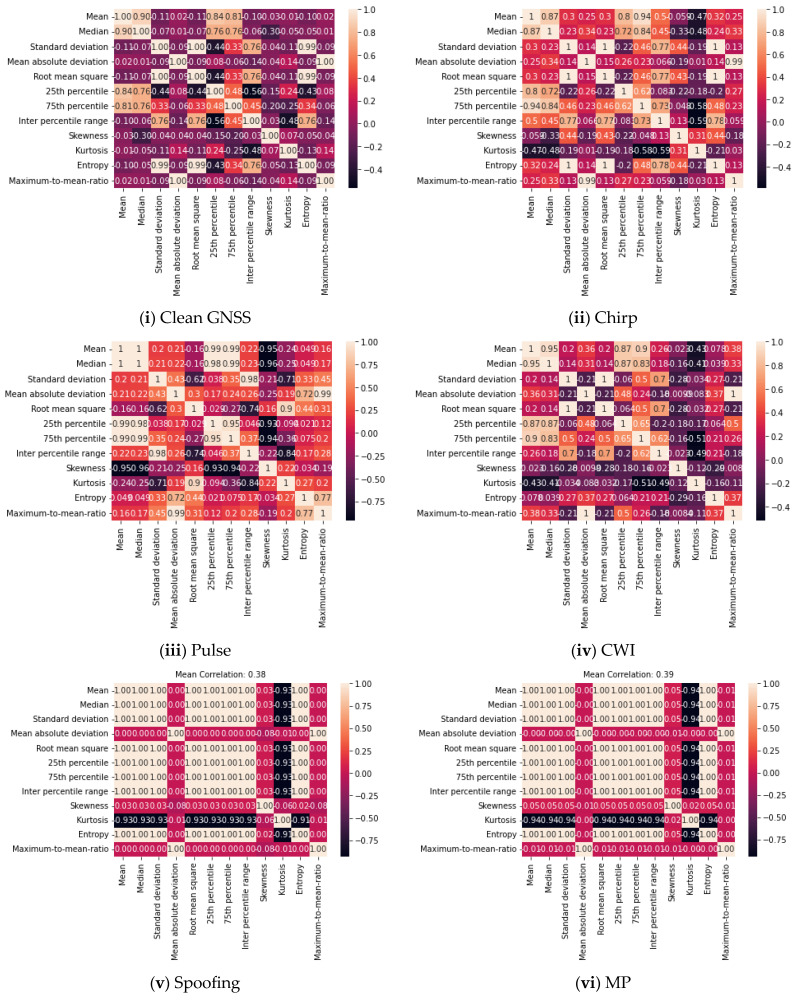

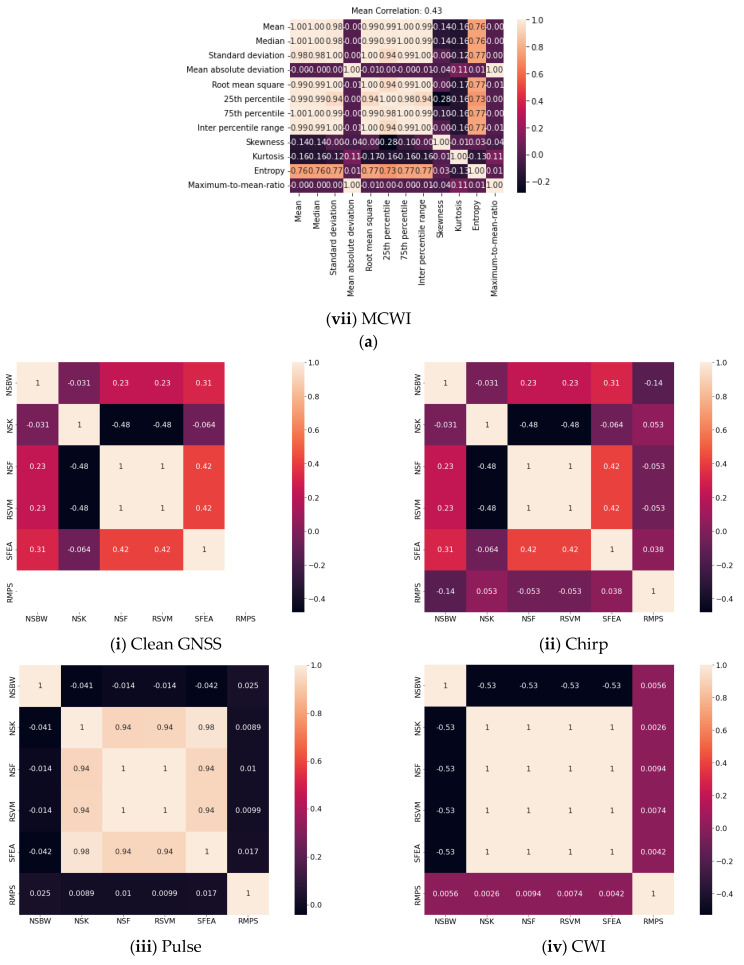

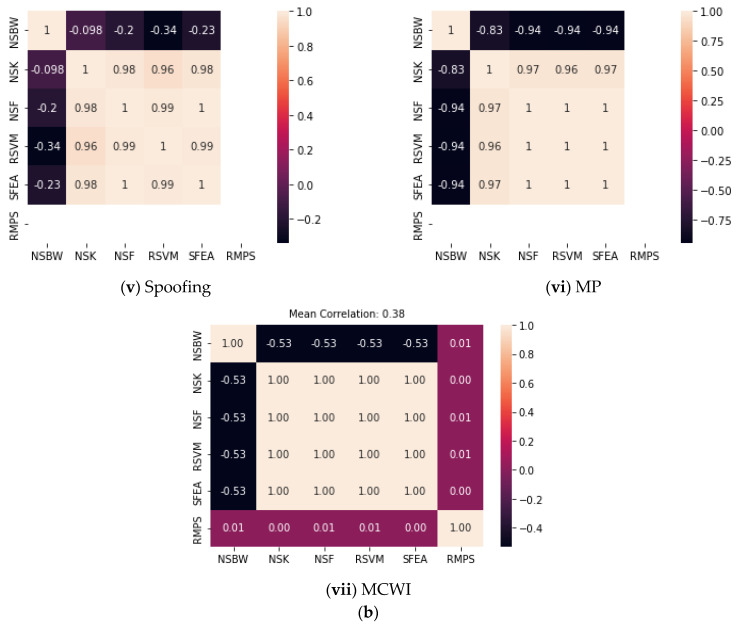
(**a**) Correlation maps for time domain features (**i**) Clean GNSS (**ii**) Chirp (**iii**) Pulse (**iv**) CWI (**v**) Spoofing (**vi**) Multipath (**vii**) MCWI. (**b**) Correlation maps for frequency domain features (**i**) Clean GNSS (**ii**) Chirp (**iii**) Pulse (**iv**) CWI (**v**) Spoofing (**vi**) Multipath (**vii**) MCWI.

When computing the feature correlation using the Pearson correlation coefficient, the closer the value is to +1, the better we can deduce from the plot that the feature being considered is the feature most correlated with the target variable (response). The correlation coefficient is defined as

(5) $$r\left(a,b\right)=\frac{\sum_{i=1}^{n}{(a}_{i}-\tilde{a}\;)(b_{i}-\tilde{b})}{\sqrt{\sum_{i=1}^{n}{{(a}_{i-}\tilde{a})}^{2}}\sqrt{\sum_{i=1}^{n}{{(b}_{i-}\tilde{b})}^{2}}},$$

where the two attribute samples are denoted by $a_{i}$ and $b_{i}$, and the mean values of the samples are given as $\tilde{a}$ and $\tilde{b}$. The correlation coefficient represents the degree of positive or negative correlation between two variables, with its value ranging from −1.0 to +1.0. the darker colours signify weaker correlations, while the lighter shades denote stronger correlations. Figure 3a,b show the correlation maps of the weakly and strongly correlated features of the deteriorated GNSS signal in both domains.

**Figure 4:**
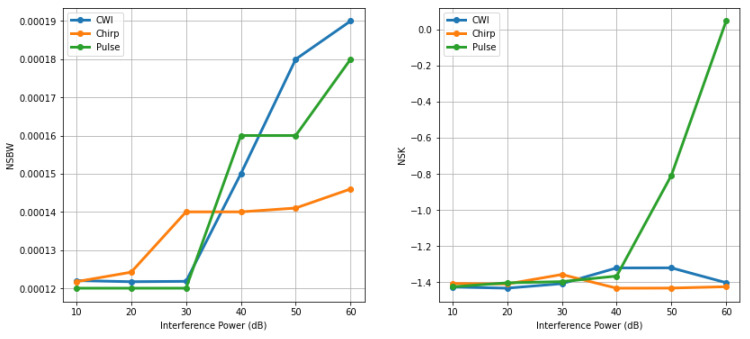

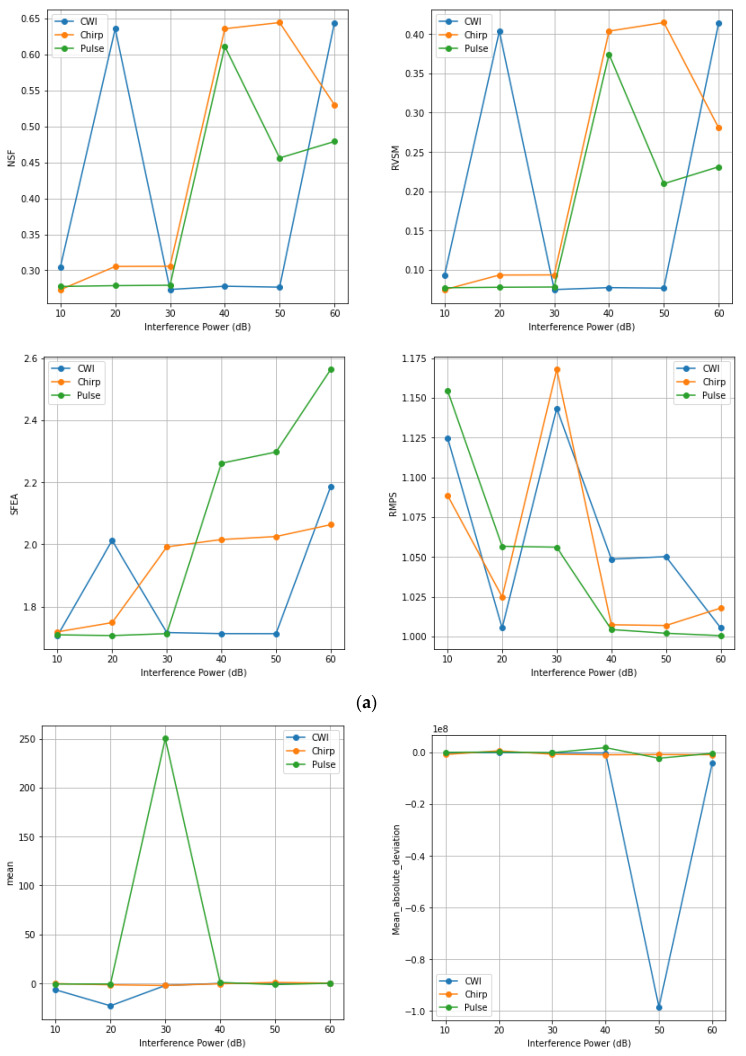

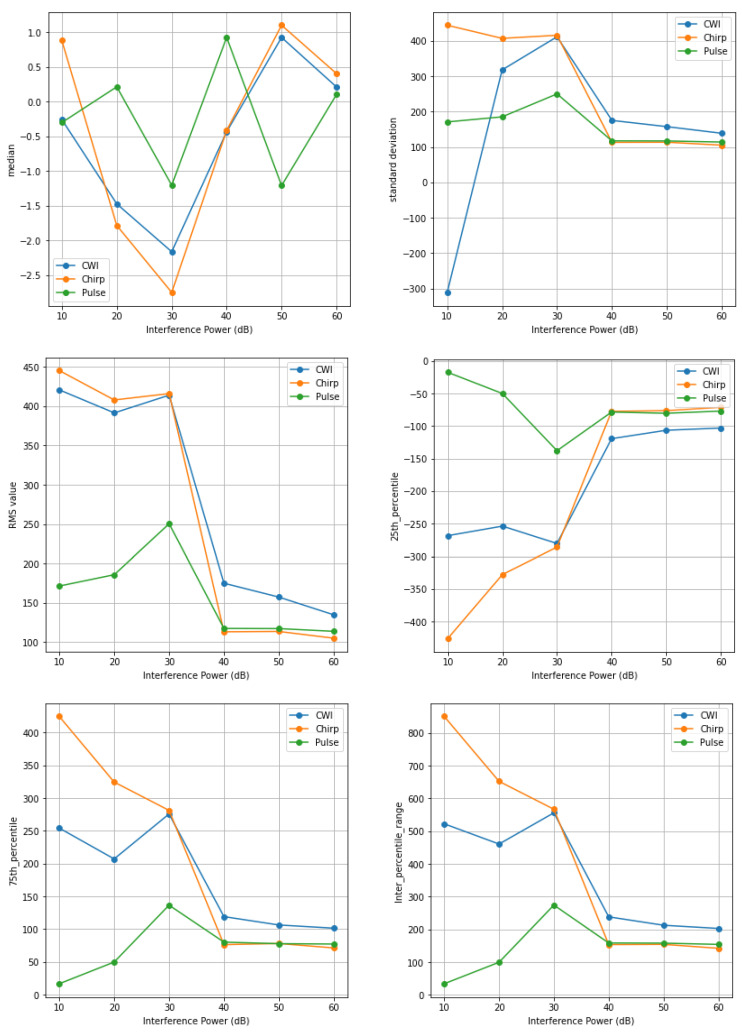

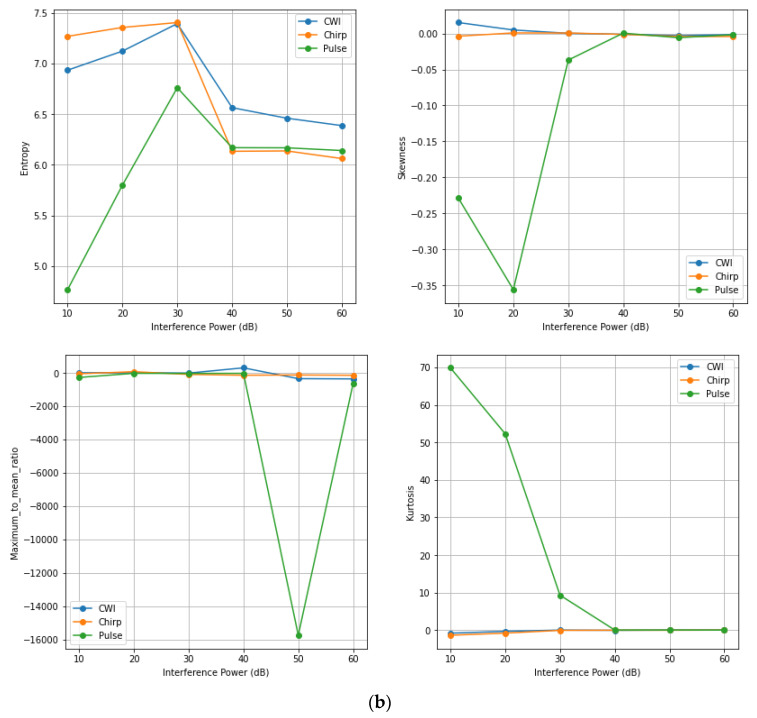
(**a**) Frequency domain features at different jamming power levels, (**b**) Time domain features under various jamming power levels.

Figure 4a illustrates the power levels of different types of interference versus six frequency domain features. Notably, as the power level increases from 40 to 60 dB, there is a sudden rise in the normalized spectral kurtosis value. For chirp-type interference, the normalized spectral flatness, RVSM, and RMPS values are slightly elevated at mid-range interference power levels.

In contrast, the frequency domain feature, depicted in Figure 4b, shows a spurious peak in the mean value for pulse-type interference around a 30 dB interference power level. Additionally, features such as the Root Mean Square (RMS), 75th percentile, inter-percentile range, entropy, and skewness exhibit a reduction in values compared to other types of interference. The kurtosis value for pulse-type interference varies significantly, whereas it remains constant for other types of interference.

**Figure 5:**
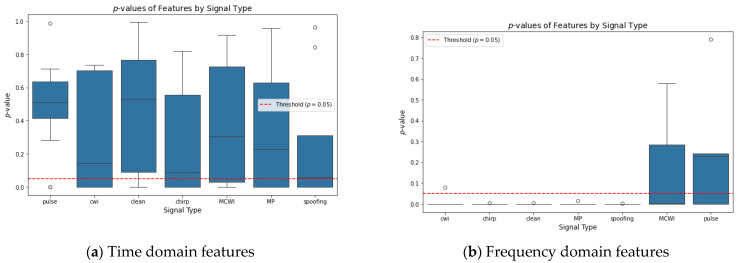
Boxplot comparison of different disruptions in GNSS signals based on *p*-values.

The next step was to decide on the best choice among two signal domain attributes, primarily depending on how well each set of features could distinguish between the different signal types. The statistical analysis has been made for both time and frequency domain attributes of various GNSS signal categories. We conducted a *p*-test to evaluate the normality of the signals. The null hypothesis of the *p*-test of the residuals follows a normal distribution. Usually, the hypothesis is rejected if a *p*-value is less than 0.05. To visualize the results, a box-and-whisker plot of the *p*-values is plotted in Figure 5 for 25.6 s of data for each signal.

The black line of the box-and-whisker plot represents the median *p*-value that indicates the degree of normality. Based on the level of significance, the *p*-value is set to 0.05, denoted by a red dashed line. This confirms a high likelihood chance that the distribution is not normal below this value. The *p*-value is greater than 0.05, revealing that the residuals tend to follow a normal distribution. It is observed, based on the analysis, that the time domain attributes generally behave as normal distributions, and it is very difficult to find significant deviations. Therefore, the time domain attribute may not be an ideal choice for analyzing GNSS signal disruption using ML techniques. On the other hand, the *p*-values in the frequency domain for most GNSS signal categories were below 0.05 except for pulse, indicating deviations from normality and providing more promising information for analysis.

## 5. XAI Analysis for GNSS Signal **Disruption** Classification

### 5.1. Data Preparation

The dataset recorded from the Skydel simulator containing I and Q raw data was processed based on user-defined specifications from the RF front end. The samples were then prepared in both domains: the time domain and the frequency domain (by computing the FFT). As described in the previous section, the statistical features were generated and pre-processed (removing the missing values and duplicate records). The resulting dataset was subsequently partitioned into training and testing datasets.

### 5.2. ML Algorithm Training and Testing

**Figure 6:**
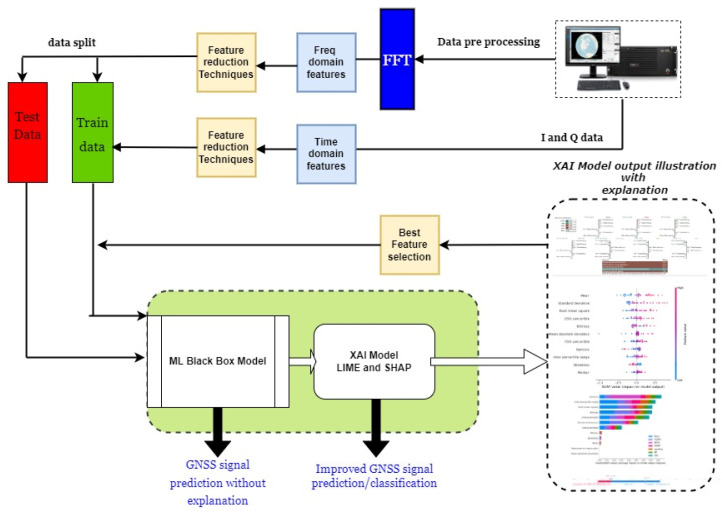
The proposed XAI framework-based GNSS signal disruption classification model.

The proposed model architecture for GNSS signal abnormality classification is depicted in Figure 6. In this work, five types of ML black-box models were employed to classify different types of signal disruptions in GNSS signals. Alongside these, a model explanation framework using LIME and SHAP was developed in parallel, with the sophisticated capabilities of this model explanation being feature contributions, an importance plot, a SHAP force plot, LIME model explanations, the best-suited features only being selected for training, and the redundant features and least important features being eliminated. The evaluation metrics are compared with traditional feature selection and elimination methods Principle Component Analysis (PCA, backward and forward selection methods). The redundant and unimportant features are neglected, and only the most important features are chosen for training. Then, based on the evaluation metrics (accuracy, precision, recall, F1-score, and Receiver Operating Characteristic ROC), the best-performing ML model was selected. Finally, knowing the cause of not predicting a particular class and the significance of the most trustworthy features for predicting the undesirable event would be helpful in analyzing the catastrophic failures in the GNSS ground-station data collection system so that the end user can take precautionary measures based on where exactly the signal quality is degraded and what factors contribute to its abnormal behaviour in the earlier stage before it is subjected to the post-processing stage. The ML models alone failed to capture the relationship between the features when it came to predicting abnormal behaviours in adverse conditions under jamming and spoofing signal attacks. However, this XAI framework helped to identify the irregularities, making it possible to investigate and respond quickly based on the explanations associated with the features in safety-critical operations.

The collected GNSS data have a duration of 40 s. A training-validation-testing split of 64-16-20% is chosen in this work. Each millisecond of data consists of 32,676 samples. We have used 25.6 s of data for training, which is 80% of 32 s of data, and 6.4 s of data are used for the validation phase. The remaining 20% of the total data, with a duration of 8 s, are used for the testing phase.

### 5.3. Local Explanations Results—LIME Technique

**Figure 7:**
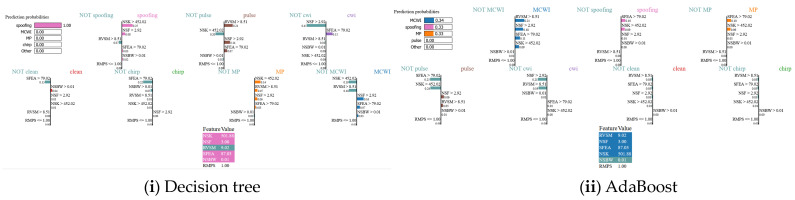

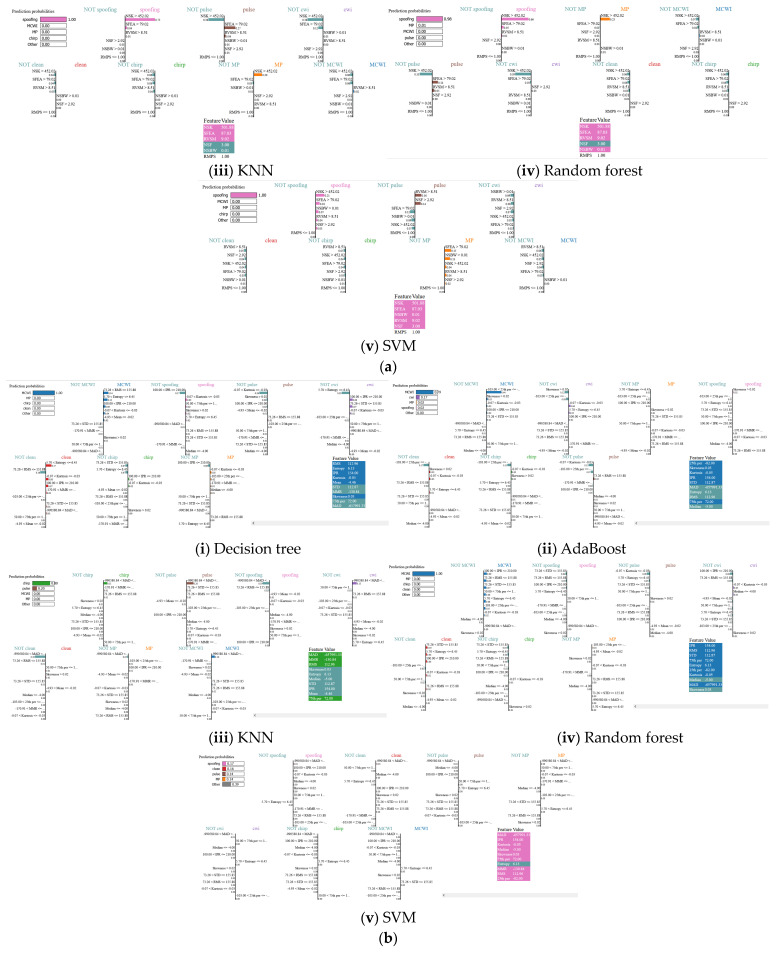
(**a**) Frequency domain LIME model decision for correct/incorrect predictions of GNSS signal for different ML models (**i**) Decision tree, (**ii**) AdaBoost, (**iii**) KNN, (**iv**) Random forest, and (**v**) SVM. (**b**) Time domain LIME model decision of correct/incorrect predictions of GNSS signal for different ML models: (**i**) Decision tree, (**ii**) AdaBoost, (**iii**) KNN, (**iv**) Random forest, and (**v**) SVM.

The prediction outputs of various ML algorithms are analyzed using the LIME model, which generates the local explanations by approximating the ML model’s output with an interpretable result based on the class prediction probabilities. The explanations for the predictions of the DT, KNN, AdaBoost, RF, and SVM models using the frequency domain features are shown in Figure 7a. For a specific sample in the test dataset, the model correctly predicted the class as spoofing, as the most important features for this sample are ‘normalized spectrum kurtosis’ and ‘single-frequency energy aggregation’ have more positive values that are the reason why the model correctly predicted the sample as spoofing. From the results, it is evident that the total value of features confirming that it is a spoofing category is greater than that of non-spoofing features. Therefore, the model correctly predicted this sample as spoofing. On the other hand, using the AdaBoost algorithm, the prediction probability of the testing instance appears to be 33% for MP and MCWI; this ambiguity led to inaccurate results despite the sample belonging to the spoofing category. The performance metrics of ML models using the LIME framework are given in Table 1a. Among all models, the SVM has the highest prediction results, and AdaBoost showed poor performance.

The prediction results of choosing all 12 features in the time domain using LIME are shown in Figure 7b. The RF model has the highest prediction accuracy of 71%, followed by DT and SVM. These models correctly predicted the class as MCWI, whereas other ML models could not be able to give the correct prediction. However, compared to frequency domain attributes, the performance of the ML model is significantly lower because most of the time, domain characteristics of the signal make it very difficult to distinguish the temporal relationships across the categories such as the multipath, spoofing, and clean signals, as these signal characteristics are highly similar in the time domain.

Table 2a shows the ML models’ performances based on the predicted label on the n^th^ instance for the corresponding test sample using all six features in the dataset using the LIME algorithm. Here, there is difficulty in predicting the clean signal, as can be seen from the outputs of all the ML models. The AdaBoost model could not predict the MP, MCWI, clean, and spoofing signals correctly and failed in predicting all instances. RF has superior performance in predicting all kinds of interference, except chirp. Compared to all models, the SVM provided better metrics.

Table 2b gives a quantitative assessment of the ML model performance on LIME, which is essential for comparing the effectiveness across various scenarios. When it comes to the time domain features, the DT and RF models performed exceptionally well; in fact, the robustness and reliability of the model results can be seen in several cases with a perfect score of 100%. However, looking into the KNN and SVM models showed significant variability in some conditions; they also achieved very low accuracy, recall, and F1-score values. AdaBoost completely failed to provide the correct signal prediction for the MP and spoofing signals but performed well in other categories.

### 5.4. Local Explanations Results—SHAP Technique

SHAP is a black-box mathematical model revealer that can uncover the hidden operations in the outcome of any ML model. The explanation of the output of the ML model is governed by the game theory concept; it involves finding each feature’s contribution to the prediction. This method provides interpretability and explainability in both formats: global and local contexts. The Shapley value, which is fundamental to this approach, reflects the average expected marginal contribution of a feature across all possible feature combinations.

The impact of the most significant feature and its contribution to the prediction is governed by the following equation [34]:

(6) $$\varphi_{j}\left(val\right)=\sum_{S\subseteq \{x_{1},\ldots ..x_{p}\}\backslash \{x_{j}\}}\left|S\right|!\frac{\left(p-\left|S\right|-1\right)!}{p!}\;\left(val\;\left(S\cup \{x_{j}\}\right)-val(S)\right)$$

The term $\varphi_{j}\;$∈ R represents the Shapley value for feature j. *S* ∈ {0, 1} denotes the subset of features included in the model, *x* is the vector of feature values for the instance being explained, *p* is the total number of features, *val*(*S*) is the prediction for the feature values in set *S*, marginalized over the features not included in *S*.

**Figure 8:**
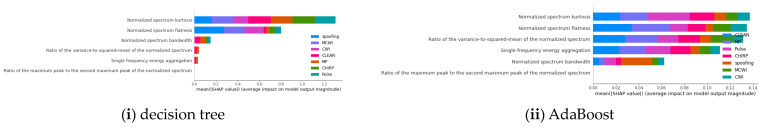

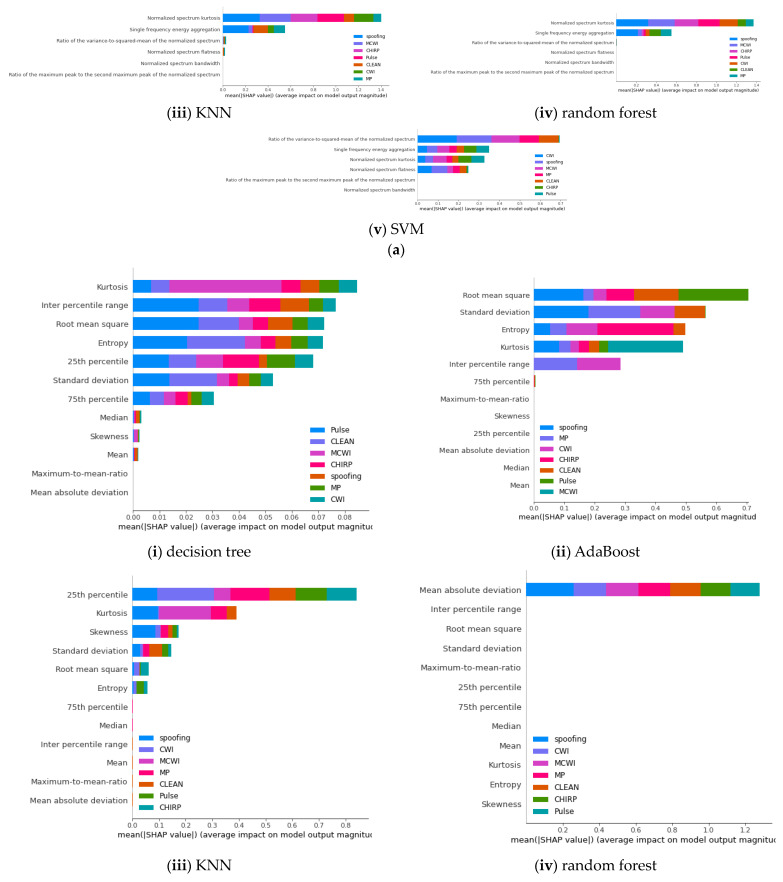

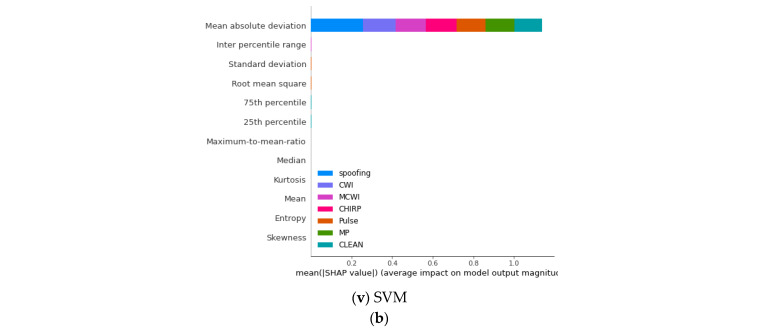
(**a**) Feature contributions based on frequency domain attributes: (**i**) decision tree, (**ii**) AdaBoost, (**iii**) KNN, (**iv**) random forest, and (**v**) SVM. (**b**) Plots of contributions of combinations of time domain features for different types of GNSS signal disruptions: (**i**) decision tree, (**ii**) AdaBoost, (**iii**) KNN, (**iv**) random forest, and (**v**) SVM.

The XAI method is applied to all ML models, and based on the SHAP algorithm, the prediction of each class is computed; the influences of these individual features are plotted in ascending order in Figure 8a. For the frequency domain features, the normalized spectral kurtosis has provided the dominant value, followed by single-frequency energy aggregation, and the least important features are identified as the ratio of the minimum peak to the maximum normalized peak and the normalized spectral bandwidth. Similarly, for the time domain attributes, entropy and the root mean square error have played a significant role in predicting the output classes. The less important features are the skewness, median, maximum-to-mean ratio, and mean absolute deviation, which contributed very little to the prediction. A feature ranking based on the relative importance is given in Table 3a.

The SHAP value of the RF model’s output explains how the time and frequency domain features impact the output of the model. In Figure 8b, the impacts of the features on the GNSS signal disruption classes are stacked to create a feature importance plot. The summary plot for multiclass classification illustrates what the XAI model has learned from these features.

While analyzing the frequency domain features, except for KNN, RF, and other ML algorithms, the prediction of the model is determined by more than three features. For a clean signal, spectral flatness contributes the most. In a multipath signal, all features contribute equally except the normalized spectrum bandwidth and the ratio of the maximum peak to the second maximum peak of the normalized spectrum. For a pulse signal, spectral kurtosis is the most significant contributor. The least important feature is identified as the normalized spectrum bandwidth for the spoofing category. The plot shows that the normalized spectrum bandwidth has a greater contribution compared to other features. In both the CWI and MCWI cases, all features contribute equally, except the normalized spectrum bandwidth.

Table 3b shows the feature ranking based on the SHAP algorithm. The majority of features are ranked between fourth and tenth; therefore, there is no combined effect of picking the best-matched features after fine-tuning the ML models’ hyperparameters (DT: maximum depth from 4–50, minimum leaf samples from 5–20; RF: maximum depth from 5–50, number of estimators from 50–150; KNN: no. of neighbours from 4–10, leaf size from 5–50; SVM: regularization parameters 1–10, radial basis function, linear kernel; AdaBoost: depth from 5–50). Selecting the dominating attributes collectively is unreliable for the time domain features since every machine learning model generates a distinct rating.

**Figure 9:**
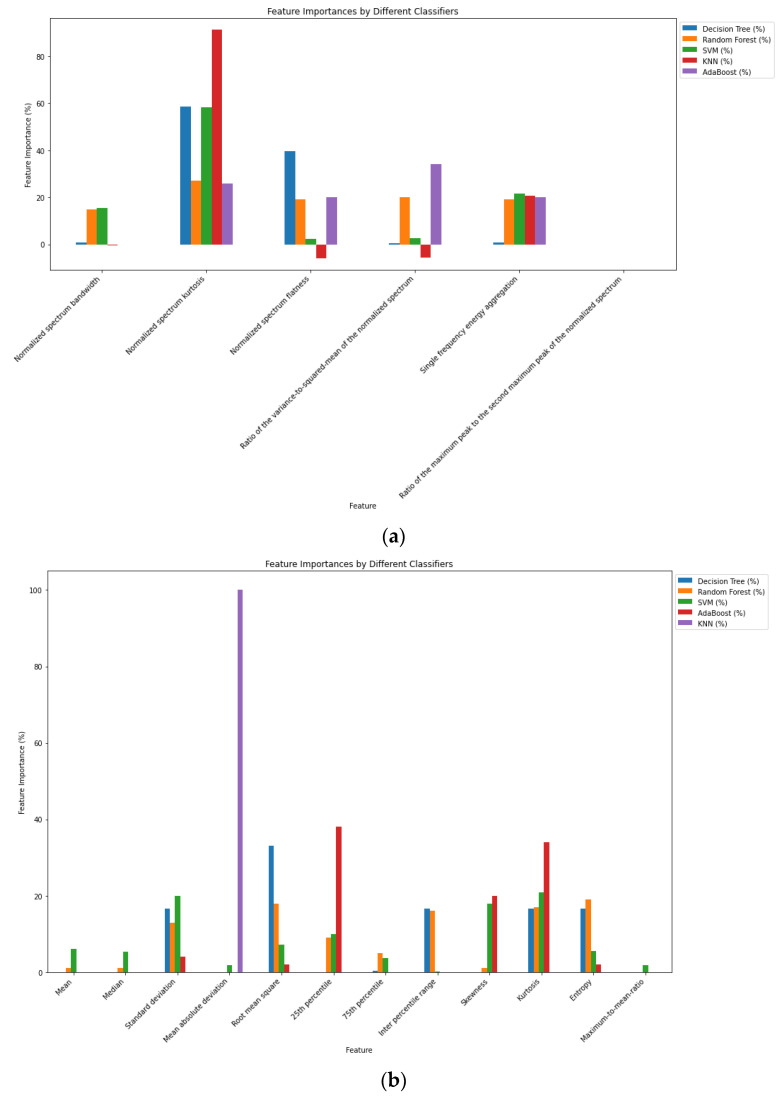
(**a**): Frequency domain feature importance comparison using different ML algorithms. (**b**): Time domain feature importance comparison using different ML algorithms.

Based on the order of attributes, the time domain feature importance plot is shown; we can see that the class CWI hardly uses the median, skewness, mean, max.-to-mean ratio, and mean absolute deviation. Additionally, the pulse and chirp classes use the same features. Kurtosis contributed significantly to the MCWI signal, which is why confusion arises between these classes relatively often. In order to separate the MP and clean signal types in a better way, new features need to be generated that are uniquely dedicated to distinguishing these classes. The normalized spectrum kurtosis has contributed significantly more, followed by single-frequency energy aggregation. For testing on all ML models, the ratio of the maximum peak to the second maximum peak of the normalized spectrum did not show much importance at all. For the time domain features, the mean, median, and maximum-to-mean ratio also do not contribute to the model’s prediction. A comparison of all models in both the time and frequency domains is depicted in Figure 9a,b.

The mean, median, 75th percentile and maximum-to-mean ratio are the least important features, and most of the attributes for ML models in the time domain are not utilized to predict the output. The mean absolute deviation is the only feature used in KNN. In the frequency domain, the normalized spectrum kurtosis is used by most of the ML models, as shown earlier in the feature importance plot; also, the ratio of the maximum peak to the second maximum peak of the normalized spectrum has not been considered much in forecasting the results of the model.

In this work, initially, the global SHAP values for all ML models are calculated; then, the feature importance is computed by multiplying the SHAP value with the model accuracy for each feature. Finally, we determine the overall average ranking across models. The frequency domain feature values are chosen from the initial value for the top k features. The metrics of all ML models show that a suitable performance can be obtained by selecting only the top four features and removing two unimportant features, resulting in the same performance, as can be seen in Table 4a. There is not much improvement in choosing more than four attributes; therefore, the last two features did not considerably improve the model performance.

Similarly, the time domain features are also used to evaluate the model’s performance, and there is no change in the accuracy value after increasing the feature values beyond K = 6; the RF model gives a good performance compared to the other models. Table 4b shows the effectiveness of top features in classifying the types of disruption. For the most important features, using traditional feature engineering, the model behaviour is not interpreted with respect to the types of disruption.

**Figure 10:**
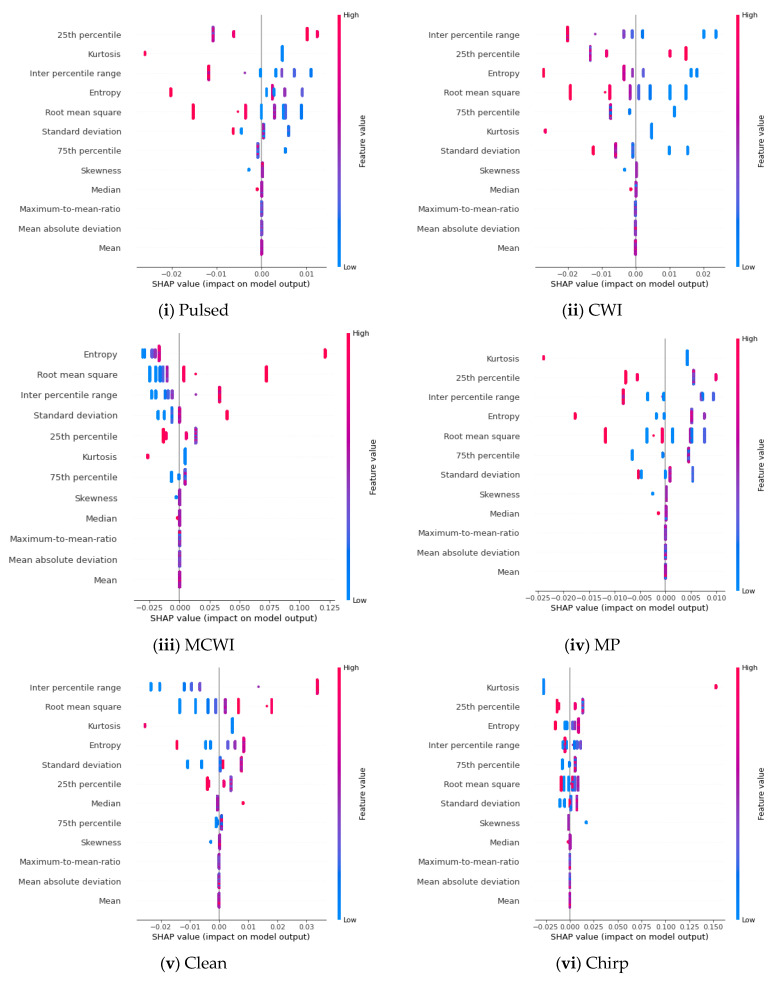

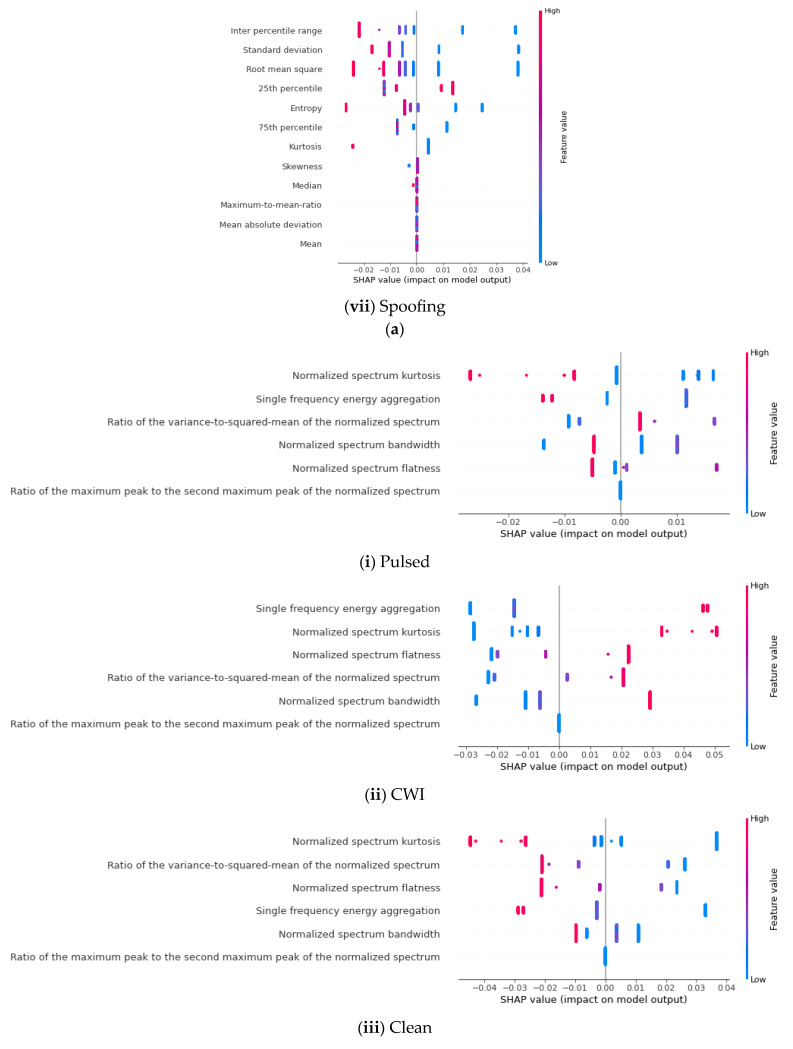

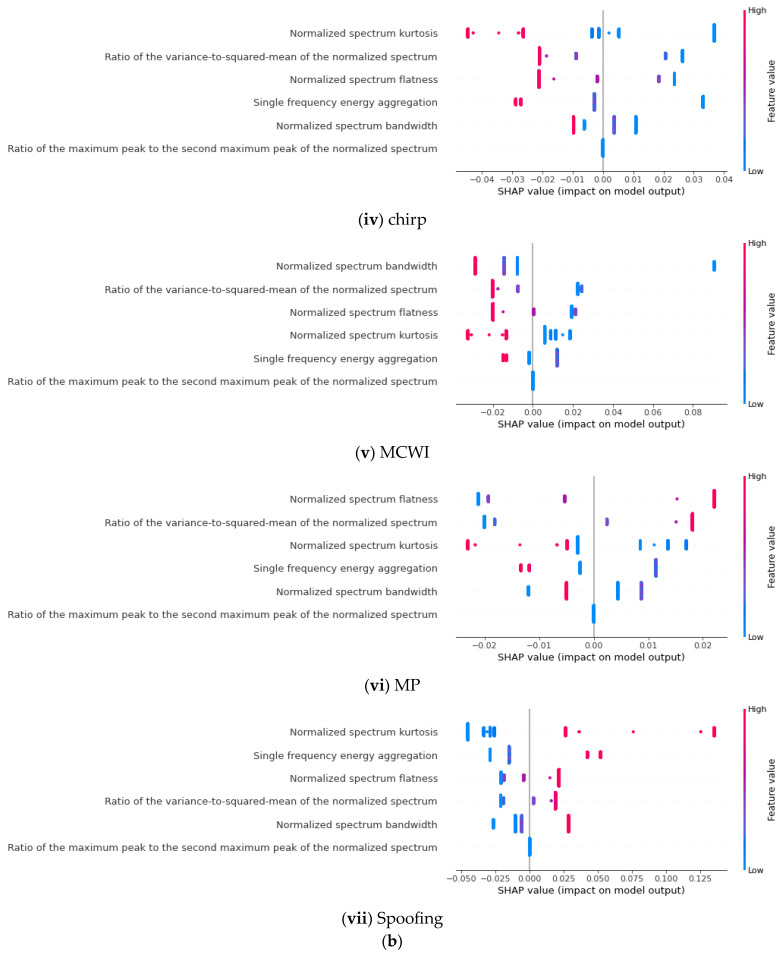
(**a**) Waterfall summary plots (time domain), (**i**) Pulsed; (**ii**) CWI; (**iii**) MCWI; (**iv**) MP; (**v**) Clean; (**vi**) Chirp; (**vii**) Spoofing. (**b**) Waterfall summary plots (frequency domain), (**i**) Pulsed (**ii**) CWI; (**iii**) Clean; (**iv**) chirp; (**v**) MCWI; (**vi**) MP; (**vii**) Spoofing.

The features are ordered by their importance for each category of jamming instances using the summary plot in order to jointly analyze the feature importance and feature effects. Each point represents a Shapley value for a feature and an instance. The position on the y-axis corresponds to the attributes, and the x-axis specifies the Shapley value, with the colours indicating the feature values from low (blue) to high (red). A positive value pushes the prediction value higher, and negative values lower the prediction. The summary plots in Figure 10a show that the mean absolute deviation, being the least important feature, has low Shapley values for CWI and chirp interference. For MP and spoofing, the following features have no impact: the mean, mean absolute deviation, maximum-to-mean ratio, median, and skewness.

In Figure 10b, the summary plot illustrates that the least significant feature is the RMPS, with low Shapley values, for the CWI and spoofing categories. The chirp interference summary plot indicates that single-frequency energy aggregation and normalized spectrum kurtosis have a more neutral effect on the model’s predictions, and features like the normalized spectrum bandwidth and the ratio of the variance to the squared mean of the normalized spectrum have a significant impact. The dispersion of points also shows how different features have an impact on various cases.

**Figure 11:**
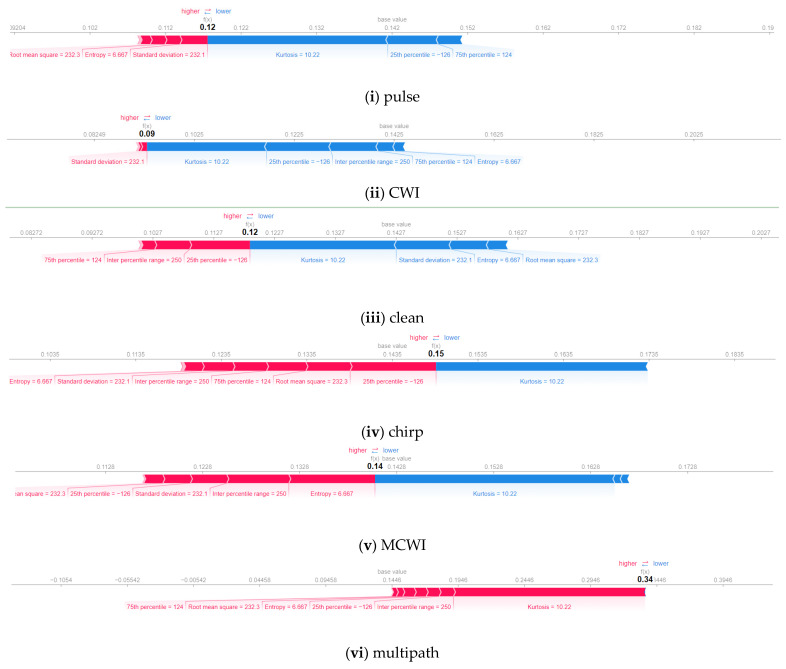

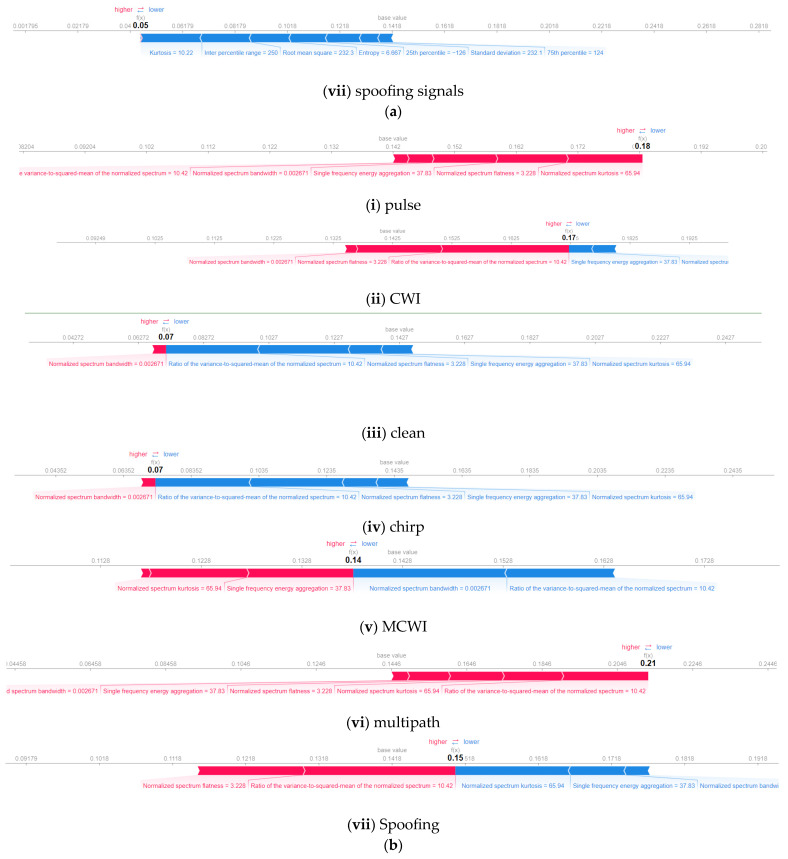
(**a**) SHAP force plots for GNSS time domain features, From top: (**i**) pulse, (**ii**) CWI, (**iii**) clean, (**iv**) chirp, (**v**) MCWI, (**vi**) multipath, and (**vii**) spoofing signals. (**b**) SHAP force plots for GNSS frequency domain features. From top: (**i**) pulse, (**ii**) CWI, (**iii**) clean, (**iv**) chirp, (**v**) MCWI, (**vi**) multipath, and (**vii**) spoofing signals.

The outcome of a SHAP model with a clear explanation for a specific GNSS signal type is visualized in Figure 11a,b in the form of a force plot. An illustration of a signal model prediction is displayed here, identifying the individual features’ contributions and providing an error analysis of signal disruption. The base value is fixed as 0.142, and the predicted value comes closer by 0.12 for a pulse signal, where the standard deviation had higher values, and the rest of the features had much lower values. For chirp and MCWI, kurtosis is a common feature that pushed the model to a lower score. In the case of MP, all features pushed in the positive direction, but kurtosis played a dominant role in correct prediction. Conversely, for the spoofing signals, most of the features are located below the base value of 0.148, pushing the model toward a lower prediction value of 0.05. The force plot for chirp interference reveals that it significantly pushes the prediction higher with a contribution of +0.15, while kurtosis reduces it by −0.0235, resulting in a final prediction of 0.1735. Similarly, in the MCWI case, kurtosis also contributed very little. In contrast, most of the features help to predict the multipath instance, and none of the features are important for predicting the spoofing instance.

The pulse signal prediction is based on all strongly correlated features that are above the base value of 0.142, whereas for the CWI signal, only two features (single-frequency energy aggregation and normalized spectral kurtosis) lie above the base value of 0.1425. The clean and chirp signals are similar to each other, with the normalized spectral kurtosis pushing the model to a higher score. Most of the features contributed to MP prediction since all predicted values are above the base value to yield the correct occurrence.

The One-vs-the-Rest (OvR) multiclass technique, commonly referred to as one-vs-all, is used to compute the area under the curve (AUC) for all machine learning models. To do this, an ROC curve must be computed for every GNSS signal class. In each iteration, one class is considered the positive class, and the other is regarded as the negative class. In the combined way, the classes are framed, and to consider every class equally, the metrics for each class are determined separately, and finally, we average them. By using these steps, the false positive and true positive rates of each class are aggregated based on the following equations:

(7) $$TPR=\sum_{i}\frac{{TP}_{i}}{{TP}_{i}+{FN}_{i}},$$

(8) $$FPR=\sum_{i}\frac{{FP}_{i}}{{FP}_{i}+{TN}_{i}}.$$

**Figure 12:**
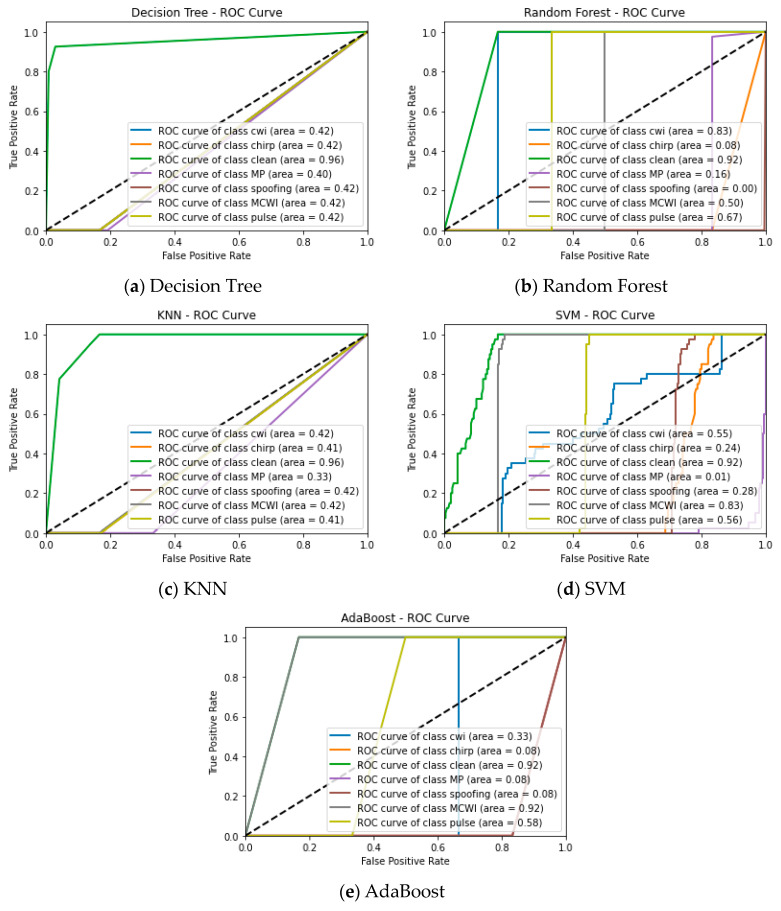
ROC one-vs-rest multiclass plot of machine learning models tested under different GNSS signal disruptions.

The KNN performs best in detecting clean signals by plotting the ROC curve with a score of 96, whereas other ML models such as RF, SVM, and AdaBoost performs closely around 90 while the DT lags with 42. For CWI detection, RF shows superior performance with 83, the SVM provides marginal performance around 55, and AdaBoost is the lowest at 33. The model’s ability is determined based on the area under the curve and how the model distinguishes between different classification categories, as shown by the ROC curve in Figure 12. The clean signal has occupied the highest area for most of the ML algorithms. For the AdaBoost, RF, and SVM algorithms, the model assigned a lower probability to the positive values, flipping the labels of the negative class and resulting in a lower area for the categories of MP and spoofing. The ROC plots for all categories are shown in Figure 12.

**Figure 13:**
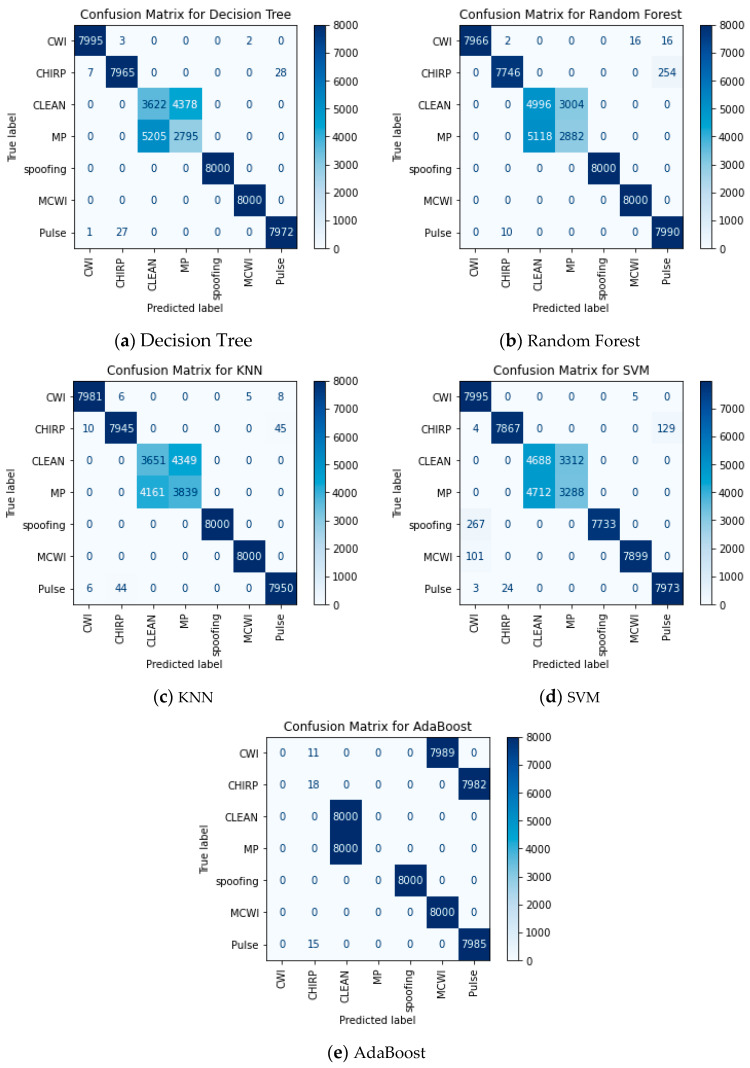
Confusion matrices (frequency domain features).

As per the frequency domain features, the classification of signal disruptions is evaluated in the form of a confusion matrix, as shown in Figure 13. The AdaBoost algorithm completely misclassifies the clean and multipath signals. The occurrence of this false classification between the MP and clean signals is due to similar kinds of signal characteristics in the frequency domain features, as the model should be able to capture any dissimilarities in the spectra of both disruptions. This instance is handled using the DT, Random Forest, KNN and SVM algorithms with more than 40% error.

Table 5 shows the results of feature selection using SHAP compared with other traditional feature selection methods; here, the optimal ‘k’ feature value is chosen as 4, based on the top features that are more trustworthy in classifying the instances correctly. Using the SVM method, SHAP has shown the leading performance when classifying the different GNSS signal disruptions. Other than the chirp signal, most of the signals are correctly classified, but when other feature selection/elimination methods are applied, the clean and spoofing signals are not properly classified. The superiority of the SHAP-based novel feature selection method can be seen in Table 5. The DT and KNN ML models, for most of the labels, classify the signal correctly, but when traditional methods are applied, the performance metrics are lower. For RF and KNN, our feature selection methods show the best performance, along with other feature reduction methods. Finally, for AdaBoost, the overall accuracy decreases under our methods; however, other performance metrics (precision, recall, and F1-score) increase for detecting the abnormalities in the signal classification.

## 6. Discussion

### Major Findings and Future Scope

The study discussed in this paper primarily focused on the effectiveness and interpretability of employing ML algorithms and using XAI techniques for GNSS signal classification and detection tasks. The results demonstrated that ML models performed at the average level for signal classification; however, the decision-making process of the model was entirely dependent on interpretability and finding the inner relationship between the features. By incorporating the LIME and SHAP algorithms into feature importance and model predictions, improved transparency and easier model understanding can be achieved. Furthermore, the research findings demonstrated that the model’s prediction effectively leveraged domain knowledge and created causal relationships, leading to an enhanced comprehension of the attributes influencing abnormal behaviour in the data. Overall, the results showed that anomaly detection can be performed in a better way, and the system has the ability to grasp different behaviours in the data when the ML algorithms are combined with explainability techniques.

The major challenges in classifying the GNSS signals captured in a real-time scenario were that it was completely different from the simulation environment, and the signal reception could be completely deteriorated by narrow-band and wide-band interference [5]. Furthermore, in an urban short-delay MP environment, from analyzing the time domain characteristics, it is very difficult to understand the inner relationship between the signal attributes in the MP scenario. Similarly, the frequency domain characteristics also failed to capture the variations in the spectrum of the pure GNSS and MP categories. Analyzing characteristics from both domains may not be sufficient to identify the abnormalities in the signal. Based on the spectrogram/scalogram image features, it may be possible to find the differences concerning time and frequency observations by using advanced XAI techniques such as saliency maps or the GradCAM and EigenCAM models, which would be helpful in analyzing the variations in the signal behaviour effectively.

## 7. Conclusions

Our research enabled an accurate classification of GNSS signal disruptions using XAI techniques on top of ML models. The significance of time and frequency domain features was considered to enhance the understanding of GNSS signal behaviour under different conditions. Moreover, accurate positioning can be achieved effectively by utilizing XAI autonomous signal quality monitoring in GNSS data processing. At an earlier stage, the root causes of an abnormality can be easily found without post-processing the signal, that is, before feeding it to the receiver. The techniques used emphasized the most discriminating part of the disruption, and the application of the XAI technique ensured transparency and interpretability in our classification, thereby taking care of trustworthiness in model development and providing confidence in the results of signal quality analysis and prediction. Overall, this study provided a robust framework for interpreting and analyzing GNSS signals. The prediction results using the LIME model show that the SVM has performed well overall, but the prediction of clean signals remains a major concern, as most of the time, these signals are incorrectly classified. Similarly, the classification results using the SHAP algorithm indicate that chirp and clean signals have the problem of misclassification, particularly between these categories. To address these challenges, careful attention needs to be paid to designing a GNSS monitoring setup equipped with sophisticated ML models that can provide more robust signal prediction/classification capabilities.

## Figures and Tables

**Table 1:** (**a**) Overall performance of the LIME model in the frequency domain using top six features. (**b**) Overall performance of LIME model on time domain using top 12 features.

(a)				
AI Model	Accuracy	Precision	Recall	F1-Score
DT	74	74	75	74
RF	75	75	75	75
KNN	79	79	79	79
SVM	81	82	81	81
AdaBoost	29	29	43	33
(**b**)				
**AI Model**	**Accuracy**	**Precision**	**Recall**	**F1-Score**
DT	66	65	64	65
RF	71	73	72	73
KNN	41	41	41	43
SVM	16	12	16	6
AdaBoost	57	48	57	50

**Table 2:** (**a**): Results of ML models’ prediction performances using frequency domain feature selection method on GNSS dataset under k = 6. (**b**): Results of ML models’ prediction performances using time domain feature selection method on GNSS dataset under k = 12.

(a)				
AI Model	Class	Precision	Recall	F1-Score
DT	MCWI	100	100	100
	MP	100	97	99
	Chirp	19	23	20
	Clean	3	3	3
	CWI	100	100	100
	Pulse	100	100	100
	Spoofing	98	100	99
RF	MCWI	100	100	100
	MP	100	97	99
	Chirp	80	70	80
	Clean	16	17	17
	CWI	100	100	100
	Pulse	100	100	100
	Spoofing	98	100	99
KNN	MCWI	100	100	100
	MP	100	97	99
	Chirp	31	35	33
	Clean	26	23	24
	CWI	100	100	100
	Pulse	100	100	100
	Spoofing	98	100	99
SVM	MCWI	91	100	95
	MP	100	97	99
	Chirp	50	68	57
	Clean	38	33	35
	CWI	100	72	84
	Pulse	100	97	99
	Spoofing	98	100	99
AdaBoost	MCWI	0	0	0
	MP	0	0	0
	Chirp	50	100	67
	Clean	0	0	0
	CWI	100	100	100
	Pulse	50	100	67
	Spoofing	0	0	0
(**b**)				
**AI Model**	**Class**	**Precision**	**Recall**	**F1-score**
DT	MCWI	100	99	100
	MP	99	100	99
	Chirp	100	100	100
	Clean	100	100	100
	CWI	100	100	100
	Pulse	100	100	100
	Spoofing	100	100	100
RF	MCWI	100	100	100
	MP	100	97	99
	Chirp	80	70	80
	Clean	16	17	17
	CWI	100	100	100
	Pulse	100	100	100
	Spoofing	98	100	99
KNN	MCWI	34	39	36
	MP	27	43	33
	Chirp	40	42	41
	Clean	26	25	26
	CWI	99	91	95
	Pulse	42	31	36
	Spoofing	30	17	22
SVM	MCWI	0	0	0
	MP	32	4	8
	Chirp	0	0	0
	Clean	28	6	9
	CWI	100	72	84
	Pulse	6	1	1
	Spoofing	98	100	99
AdaBoost	MCWI	33	99	50
	MP	0	0	0
	Chirp	4	12	14
	Clean	0	0	0
	CWI	100	100	100
	Pulse	100	100	100
	Spoofing	0	0	0

**Table 3:** (**a**) Relative frequency domain feature importance (feature ranking). (**b**) Relative time domain feature importance (feature ranking).

(a)							
Feature	DT	RF	KNN	SVM	AdaBoost	Average	Rank
Normalized spectrum kurtosis	1	1	1	1	2	1	1
Ratio of the variance to the squared mean of the normalized spectrum	6	2	4	3	1	2.6	3
Normalized spectrum flatness	2	4	3	4	4	2.83	3
Ratio of the maximum peak to the second maximum peak of the normalized spectrum	5	6	5	5	5	4.3	4
Normalized spectrum bandwidth	3	5	6	6	6	4.3	4
Single-frequency energy aggregation	4	3	2	2	3	2.3	2
(**b**)							
**Feature**	**DT**	**RF**	**KNN**	**SVM**	**AdaBoost**	**Average**	**Rank**
Mean	10	10	3	2	12	7.4	7
Median	9	9	2	3	11	6.8	7
Standard deviation	5	5	8	12	4	6.8	7
Mean absolute deviation	8	12	1	1	10	6.4	6
Root mean square error	1	2	7	4	6	4	4
25th percentile	9	6	6	6	1	5.6	6
75th percentile	6	7	4	5	9	6.2	6
Inter-percentile range	3	4	5	7	8	5.4	5
Skewness	8	8	9	8	3	7.2	7
Kurtosis	2	3	11	9	2	5.4	5
Entropy	4	1	10	10	5	6	6
Maximum-to-mean ratio	7	11	12	11	7	9.6	10

**Table 4:** (**a**) ML models’ performance results using different top ‘k’ frequency domain feature selection methods on GNSS dataset. (**b**) ML models’ performance results using different top ‘k’ time domain feature selection methods on GNSS dataset.

(a)						
No. of Features	Metric	DT	RF	KNN	SVM	AdaBoost
K = 1	Accuracy	0.4500	0.2857	0.4750	0.4607	0.4223
	Precision	0.4524	0.1667	0.5125	0.3794	0.3901
	Recall	0.4500	0.2857	0.4750	0.4607	0.4356
	F1-score	0.4504	0.1837	0.4852	0.3719	0.3421
K = 2	Accuracy	0.9750	0.7143	0.9786,	0.8250	0.5682
	Precision	0.9763	0.5656	0.9798	0.8558	0.5321
	Recall	0.9750	0.7143	0.9786	0.8250	0.5789
	F1-score	0.9749	0.6172	0.9785	0.8158	0.5971
K = 3	Accuracy	0.9867	0.8571	0.9788	0.9250	0.6210
	Precision	0.9244	0.7667	0.9425	0.9384	0.6535
	Recall	0.9462	0.8571	0.9532	0.9250	0.7152
	F1-score	0.9764	0.8023	0.9642	0.9234	0.7543
K = 4	Accuracy	0.9899	0.9672	0.9635	0.9964	0.6458
	Precision	1	0.9989	0.9785	0.9965	0.7342
	Recall	1	1	0.9826	0.9964	0.6735
	F1-score	1	1	1	0.9964	0.7211
K = 5	Accuracy	1	0.9929	1	0.9964	0.6712
	Precision	1	0.9932	1	0.9965	0.7443
	Recall	1	0.9929	1	0.9964	0.6990
	F1-score	1	0.9929	1	0.9964	0.7124
K = 6	Accuracy	1	0.8571	1	0.9250	0.6876
	Precision	1	0.7667	1	0.9384	0.7342
	Recall	1	0.8571	1	0.9250	0.6735
	F1-score	1	0.8023	1	0.9234	0.7211
(**b**)						
**No. of Features**	**Metric**	**DT**	**RF**	**KNN**	**SVM**	**AdaBoost**
K = 1	Accuracy	0.6250	0.5714	0.7036	0.4286	0.3246
	Precision	0.6252	0.4048	0.7038	0.1917	0.0869
	Recall	0.6250	0.5714	0.7036	0.4286	0.3458
	F1-score	0.6243	0.4524	0.7026	0.2633	0.3675
K = 2	Accuracy	0.4524	0.5714	0.7036	0.4571	0.4412
	Precision	0.6252	0.4048	0.7038	0.2489	0.2357
	Recall	0.6250,	0.5714	0.7036	0.4571	0.4043
	F1-score	0.6243	0.4524	0.7026	0.3205	0.3215
K = 3	Accuracy	0.7500,	0.8536	0.7786	0.7786	0.6548
	Precision	0.7489	0.7822	0.7786	0.7313	0.5436
	Recall	0.7500	0.8536	0.7786	0.7786	0.4265
	F1-score	0.7494	0.8060	0.7785	0.7262	43,445
K = 4	Accuracy	0.7480,	0.8536	0.7786	0.8250	0.6550
	Precision	0.7421	0.7822	0.7782	0.7576	0.5436
	Recall	0.7464	0.8536	0.7786	0.8250	0.4336
	F1-score	0.7439	0.8060	0.7783	0.7772	0.4987
K = 5	Accuracy	0.7480	0.8393	0.7786	0.7750	0.6548
	Precision	0.7394	0.7692	0.7588	0.7740	0.5436
	Recall	0.7464	0.8393	0.8286	0.7750	0.4265
	F1-score	0.7425	0.7916	0.7809	0.7741	43,445
K = 6	Accuracy	0.7464	0.8429	0.7750,	0.8286	0.6753
	Precision	0.7394	0.7727	0.7740	0.7588,	0.5964
	Recall	0.7464,	0.8429	0.7750	0.8286	0.4413
	F1-score	0.7425	0.7952	0.7741	0.7809	0.4842
K = 7	Accuracy	0.7464	0.8429	0.7750	0.8286	0.6759
	Precision	0.7394	0.7727	0.7740	0.7588	0.61225
	Recall	0.7464	0.8429	0.7750	0.8286	0.6709
	F1-score	0.7425	0.7952	0.7741	0.7809	0.4007
K = 8	Accuracy	0.7464	0.8429	0.7750	0.8286	0.6986
	Precision	0.7394	0.7727	0.7740	0.7588	0.6543
	Recall	0.7464	0.8429	0.7750	0.8286,	0.6709
	F1-score	0.7425	0.7952	0.7741	0.7809	0.4346
K = 9	Accuracy	0.7464	0.8429	0.7750	0.8286	0.6986
	Precision	0.7394	0.7727	0.7740	0.7588	0.6543
	Recall	0.7464	0.8429	0.7750	0.8286	0.6709
	F1-score	0.7425	0.7952	0.7741	0.7809	0.4346
K = 10	Accuracy	0.7464	0.8429	0.7750	0.8286	0.6986
	Precision	0.7394	0.7727	0.7740	0.7588	0.6543
	Recall	0.7464	0.8429	0.7750	0.8286	0.6709
	F1-score	0.7425	0.7952	0.7741	0.7809	0.4346
K = 11	Accuracy	0.7464	0.8429	0.7750	0.8286	0.6986
	Precision	0.7394	0.7727	0.7740	0.7588	0.6543
	Recall	0.7464	0.8429	0.7750	0.8286	0.6709
	F1-score	0.7425	0.7952	0.7741	0.7809	0.4346
K = 12	Accuracy	0.7464	0.8429	0.7750	0.8286	0.6986
	Precision	0.7394	0.7727	0.7740	0.7588	0.6543
	Recall	0.7464	0.8429	0.7750	0.8286	0.6709
	F1-score	0.7425	0.7741	0.7809	0.7862	0.4346

**Table 5:** Results per class for every feature selection method under k = 4.

Model	Class	SHAP				PCA				Backward Elimination				Forward Selection			
DT	Metric	Acc	Prec	Rec	F1	Acc	Prec	Rec	F1	Acc	Prec	Rec	F1	Acc	Prec	Rec	F1
	MCWI	100	100	100	100	97	95	97	92	97	95	97	92	97	95	97	92
	MP	95	100	97	99	98	89	91	90	98	89	91	90	98	89	91	90
	Chirp	24	19	23	20	21	19	21	20	23	17	32	23	25	22	26	23
	Clean	72	76	78	83	82	18	12	12	12	18	15	12	16	22	23	09
	CWI	100	100	100	100	98	88	90	93	98	88	90	93	98	88	90	83
	Pulse	100	100	100	100	97	76	81	90	97	76	81	90	97	72	80	79
	Spoofing	98	98	100	99	97	95	97	92	97	95	97	92	97	95	97	92
RF	MCWI	94	97	93	97	98	89	91	90	98	89	91	90	98	89	91	90
	MP	98	100	97	99	95	94	92	91	88	93	92	89	90	91	88	86
	Chirp	98	80	70	80	72	76	71	76	67	66	63	61	60	65	66	67
	Clean	19	16	17	17	18	13	17	15	12	13	15	12	12	13	13	11
	CWI	100	100	100	100	97	76	81	90	97	76	81	90	97	76	81	90
	Pulse	100	100	100	100	97	95	97	92	97	95	97	92	97	95	97	92
	Spoofing	98	98	100	99	98	89	91	90	98	89	91	90	98	89	91	90
KNN	MCWI	100	100	100	100	93	85	87	80	81	64	72	72	77	75	75	72
	MP	99	100	97	99	95	88	87	80	75	65	64	60	61	64	66	63
	Chirp	37	31	35	33	34	32	24	24	29	31	33	29	30	28	29	33
	Clean	27	26	23	24	32	37	31	24	19	19	24	21	20	27	32	35
	CWI	100	100	100	100	95	94	93	90	94	78	73	71	68	72	73	78
	Pulse	100	100	100	100	97	94	93	90	89	91	89	88	79	81	80	81
	Spoofing	100	98	100	99	87	97	95	93	71	75	73	74	70	71	72	71
SVM	MCWI	95	91	100	95	75	65	75	79	70	68	69	78	64	61	56	64
	MP	96	100	97	99	98	88	90	82	80	88	81	79	79	88	79	79
	Chirp	65	50	68	57	97	76	81	90	97	76	81	90	97	76	81	90
	Clean	34	38	33	35	97	95	97	92	97	95	97	92	97	95	97	92
	CWI	87	100	72	84	98	89	91	90	98	89	91	90	98	89	91	90
	Pulse	98	100	97	99	78	76	72	70	67	69	73	72	77	74	77	71
	Spoofing	97	98	100	99	84	74	75	77	86	81	71	69	73	76	77	72
AdaBoost	MCWI	25	23	26	23	24	25	24	19	22	21	24	23	27	22	24	26
	MP	45	46	45	15	44	32	37	39	42	41	32	33	39	41	40	34
	Chirp	53	50	100	67	53	54	51	43	44	35	45	32	48	42	43	35
	Clean	54	56	45	55	54	52	41	44	50	43	47	42	43	41	39	38
	CWI	56	54	56	45	45	51	42	41	44	48	50	46	52	43	39	44
	Pulse	49	50	65	67	53	54	56	47	45	56	54	50	54	52	46	46
	Spoofing	51	56	54	45	59	46	46	43	34	43	34	44	41	43	42	39

## Data Availability

The data that support the findings of this study are available upon reasonable request from the authors.
